# Cigarette smoke alters primary human bronchial epithelial cell differentiation at the air-liquid interface

**DOI:** 10.1038/srep08163

**Published:** 2015-02-02

**Authors:** Andrea C. Schamberger, Claudia A. Staab-Weijnitz, Nikica Mise-Racek, Oliver Eickelberg

**Affiliations:** 1 Comprehensive Pneumology Center, Institute of Lung Biology and Disease, University Hospital, Ludwig-Maximilians-University and Helmholtz Zentrum München, Member of the German Center for Lung Research, Max-Lebsche-Platz 31, 81377 Munich, Germany

## Abstract

The differentiated human airway epithelium consists of different cell types forming a polarized and pseudostratified epithelium. This is dramatically altered in chronic obstructive pulmonary disease (COPD), characterized by basal and goblet cell hyperplasia, and squamous cell metaplasia. The effect of cigarette smoke on human bronchial epithelial cell (HBEC) differentiation remains to be elucidated. We analysed whether cigarette smoke extract (CSE) affected primary (p)HBEC differentiation and function. pHBEC were differentiated at the air-liquid interface (ALI) and differentiation was quantified after 7, 14, 21, or 28 days by assessing acetylated tubulin, CC10, or MUC5AC for ciliated, Clara, or goblet cells, respectively. Exposure of differentiating pHBEC to CSE impaired epithelial barrier formation, as assessed by resistance measurements (TEER). Importantly, CSE exposure significantly reduced the number of ciliated cells, while it increased the number of Clara and goblet cells. CSE-dependent cell number changes were reflected by a reduction of acetylated tubulin levels, an increased expression of the basal cell marker KRT14, and increased secretion of CC10, but not by changes in transcript levels of *CC10*, *MUC5AC*, or *FOXJ1*. Our data demonstrate that cigarette smoke specifically alters the cellular composition of the airway epithelium by affecting basal cell differentiation in a post-transcriptional manner.

The differentiated human airway epithelium consists of different cell types forming a pseudostratified and polarized epithelium with about 50–70% ciliated cells^1^, 11% Clara cells^2^, up to 25% goblet cells^3^, and up to 30% basal cells^4^ in the adult airways. Undifferentiated basal cells, as well as intermediate cells, have stem cell properties and can differentiate into ciliated cells, Clara cells, or goblet cells^5^,^6^,^7^. This accounts for both normal tissue turnover as well as tissue repair in the mouse and human airway epithelium^8^,^9^,^10^. Basal cells are located just above the basement membrane and express cytokeratin 5 and 14 and the transcription factor TP63. Ciliated epithelial cells account for over 50% of all cells within the human airways and display about 300 motile cilia per cell, which stain positive for acetylated tubulin. These are important for clearing up mucus from the airways via coordinated ciliary beating. Clara cell-specific 10 kDa protein (CC10) is a major secretory protein of Clara cells with documented anti-inflammatory and immune-modulatory functions^11^,^12^. Goblet cells mainly secrete mucus, which coats the surface of the trachea and bronchioles and is a mixture of highly glycosylated mucin proteins (predominantly mucin 5AC and mucin 5B).

In chronic obstructive pulmonary disease (COPD), the airway epithelium is dramatically altered^13^. Basal and goblet cell hyperplasia, as well as squamous cell metaplasia, are reported characteristics of COPD lungs^6^,^14^,^15^. Next to environmental exposures like noxious gases, particles, indoor fumes, or pathogens, smoking is the most important risk factor to develop COPD^16^. Hence, it is of clear interest to study the contribution of cigarette smoke to the documented changes in the epithelium of COPD patients. Goblet cell hyperplasia has mainly been found in the proximal airways of (ex-) smokers with COPD^17^,^18^, but also in small airways of smokers, where goblet cells are normally absent^19^,^20^. Squamous metaplasia has been reported in proximal and distal airways of smokers and COPD patients^15^,^19^,^21^. Further smoking-associated changes include structural and functional abnormalities of ciliated cells, e.g. shortened airway cilia in smokers, which recently has been demonstrated to result from enhanced autophagy^19^,^22^,^23^. Moreover, we and others reported recently that acute exposure to cigarette smoke extract (CSE) impairs barrier function and tight junction organization *in vitro*, using primary human bronchial epithelial cells (pHBECs) differentiated at the air-liquid interface (ALI)^24^,^25^. These changes in combination with mucus overproduction and decreased mucociliary clearance promote pathogen colonization and the development of smoking-associated lung disorders, such as COPD and lung cancer. It is still unclear, however, whether the smoking-associated changes in the airway epithelium are a detrimental effect of cigarette smoke on already differentiated cells, or whether continuous cigarette smoke exposure affects basal/intermediate cell differentiation.

In order to address these questions, we differentiated pHBECs from non-diseased donors at ALI into a mucociliary epithelium in the presence or absence of CSE. Ciliated cell, Clara cell, and goblet cell numbers were evaluated during the course of differentiation up to 28 days. We found cigarette smoke-dependent alterations in the amount and function of all cell types evaluated, most importantly reduced ciliated cell numbers.

## Results

### Cigarette Smoke Extract Impairs Epithelial Barrier Function during Primary Human Bronchial Cell Differentiation

**Figure 1:**
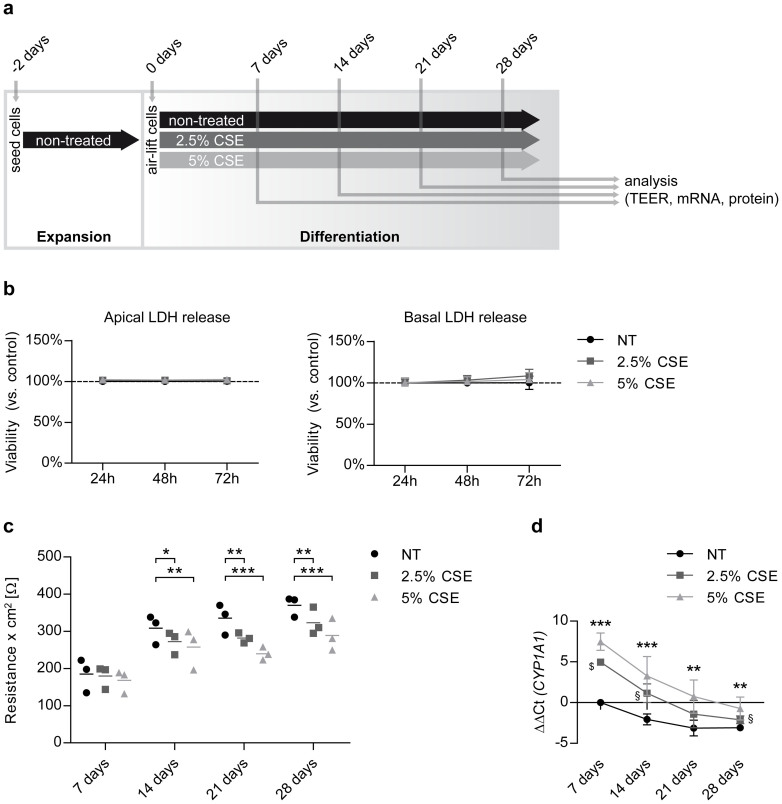
Non-toxic doses of CSE impair epithelial barrier establishment during pHBEC differentiation. (a) Schematic overview of pHBEC differentiation in the presence of CSE: pHBECs were seeded at transwell inserts and expanded for 48 hours until confluency. Cells were subsequently air-lifted and basolateral media was exchanged to differentiation medium with or without CSE. The medium was renewed every 2–3 days and samples were taken for analysis after 7, 14, 21, or 28 days of differentiation. (b) Analysis of LDH assay of air-lifted pHBECs exposed to 0% (NT), 2.5%, or 5% CSE for up to 72 hours. LDH was determined in the cell culture supernatant (left panel) as well as in the basolateral medium (right panel) from three independent experiments. Data were normalized to time-matched negative controls (non-treated cells) and positive controls (maximal LDH activity measured in non-treated lysed cells). (c) TEER development over 28 days of pHBEC differentiation in the absence or presence of CSE (2.5% or 5% CSE). Data of 3 independently performed differentiations with mean are shown. *p < 0.05, **p < 0.01, ***p < 0.001 (two-way ANOVA). (d) qRT-PCR analysis of *CYP1A1* transcript levels of pHBECs differentiated up to 28 days in the absence or presence of CSE (2.5% or 5% CSE). Data are depicted as mean ± SD from 3 independent differentiations. Relative transcript abundance of a gene is expressed as ΔΔCt = [ΔCt(gene of interest, condition NT, day 7)] – [ΔCt(gene of interest, condition X, day X)] with ΔCt = Ct(gene of interest) – Ct (reference); increase = increase in gene expression. For non-treated cells at day 21 or day 28, when transcripts levels were below detection level, Ct = 40 was used for statistical analysis. For 5% CSE vs. NT: **p < 0.01, ***p < 0.001. For 2.5% CSE vs. NT: ^§^p < 0.05, ^$^p < 0.001 (two-way ANOVA).

In order to examine the effect of cigarette smoke extract (CSE) on basal cell differentiation at the air-liquid interface (ALI), cells were exposed to CSE during the entire differentiation process and analysis was performed after 7, 14, 21, and 28 days of differentiation (Figure 1a).

During the first 72 hours, CSE concentrations of 2.5% and 5% did not trigger lactate dehydrogenase (LDH) release from the cells compared to control, neither to the apical nor the basolateral compartment (Figure 1b). Hence, these concentrations were chosen as non-toxic doses for the experimental setup. Of note, chronic exposure of differentiating pHBECs to CSE significantly decreased transepithelial electrical resistance (TEER) from day 14 on in a concentration-dependent manner (Figure 1c). 2.5% and 5% CSE highly induced cytochrome P450 1A1 (CYP1A1) transcript levels at all time points compared to control conditions, confirming CSE potency^26^ (Figure 1d). Interestingly, *CYP1A1* baseline expression significantly declined during the course of differentiation, with only limited detection at day 21 and day 28 via qRT-PCR. These data demonstrate that non-toxic doses of CSE impair the establishment of the bronchial barrier in differentiating basal cells.

### Chronic CSE Exposure Alters pHBEC Differentiation

**Figure 2:**
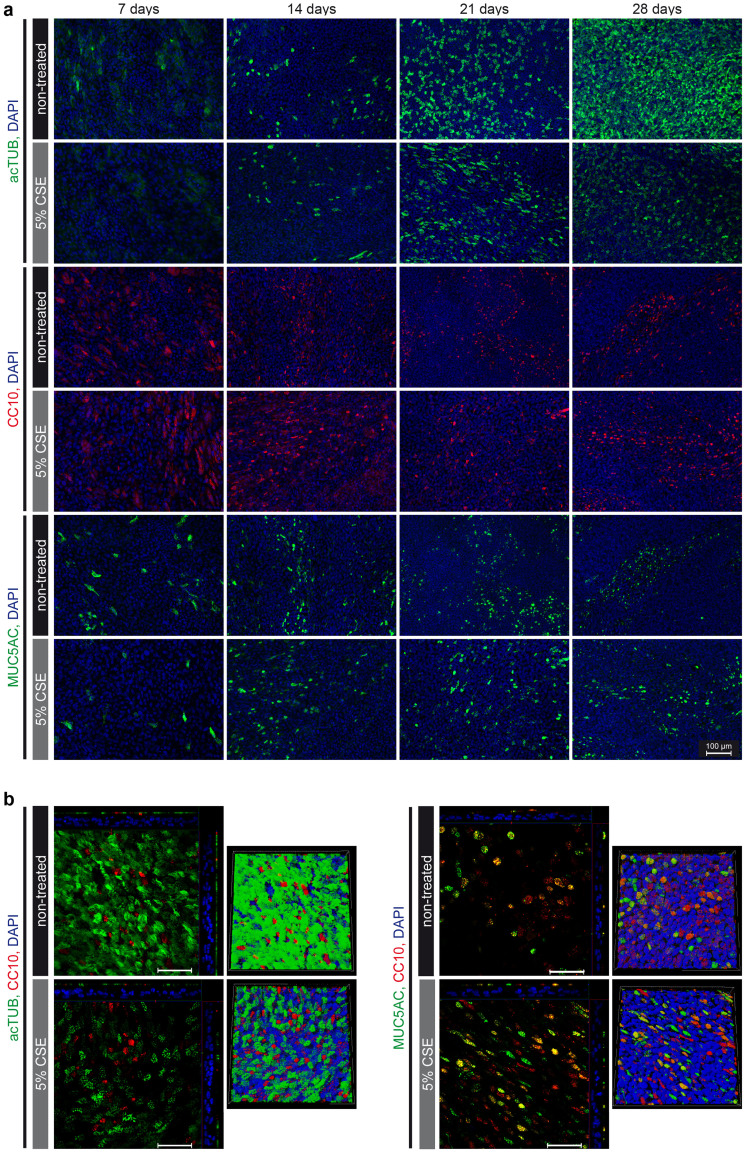
CSE specifically reduces the number of ciliated cells in differentiating pHBECs. Indirect immunofluorescence analysis of non-treated or chronically treated (5% CSE) pHBECs. (a) pHBECs differentiated for 7, 14, 21, or 28 days at ALI. Representative acTUB (acetylated tubulin), CC10 (Clara cell-specific protein), and MUC5AC (mucin 5AC) is shown in green or red, as depicted, and DAPI staining in blue. Scale bar: 100 μm. (b) Representative z-stacks (left subpanel) with corresponding shadow projection (right subpanel) are shown for 28 days differentiated pHBECs. acTUB, MUC5AC, or CC10 is shown in green or red, as depicted, and DAPI staining in blue. Scale bar: 50 μm.

**Figure 3:**
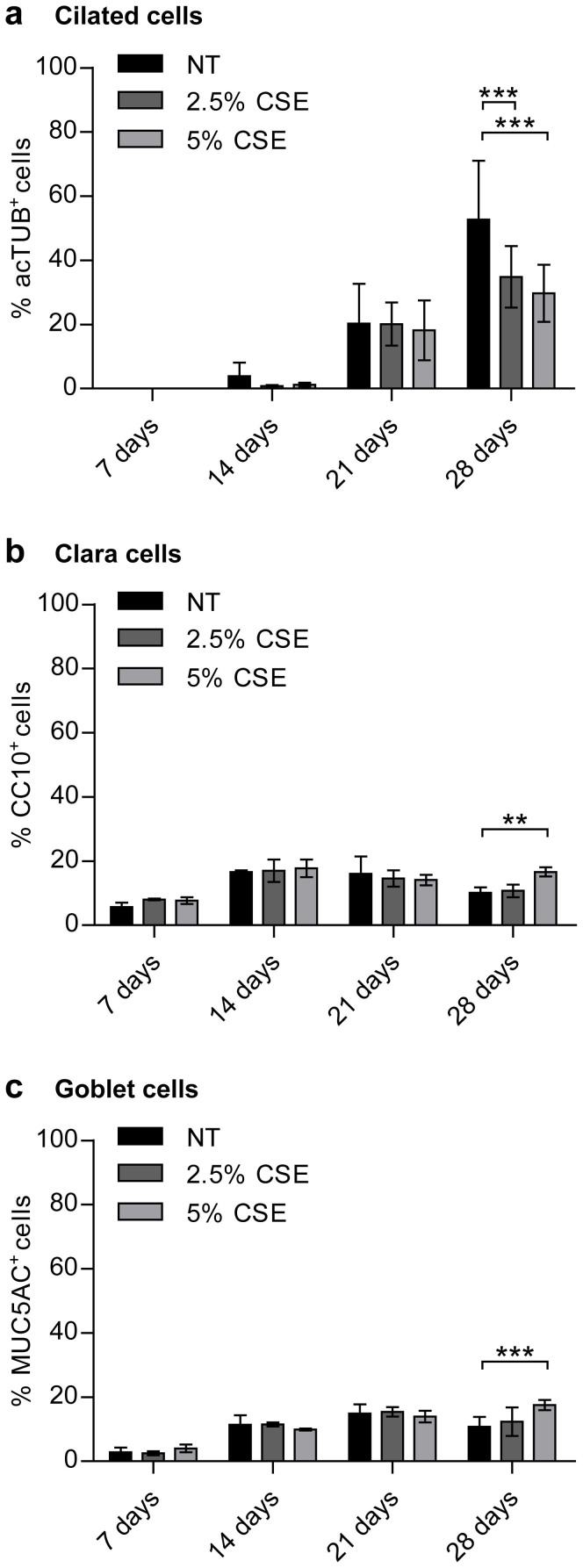
CSE shifts cell populations in differentiating pHBECs. Quantification of ciliated cells (a), Clara cells (b), or goblet cells (c) in the course of pHBEC differentiation in the absence or presence of CSE (2.5% or 5% CSE). Cell types were determined by positivity for the following markers: acTUB for ciliated cells (a), CC10 for Clara cells (b), and MUC5AC for goblet cells (c). Data are depicted as mean ± SD from 3 independent differentiations. 12 images per group were analysed. **p < 0.01, ***p < 0.001 (two-way ANOVA).

The effect of continuous CSE exposure on the emerging cell populations from basal cells differentiating at ALI was analysed by immunofluorescence analysis. For this, cells on transwell membranes were stained for cell type specific markers (Figure 2) and quantified over time (Figure 3). The number of acetylated-tubulin positive ciliated cells increased significantly over time in all cultures (Figure 2a upper panel and Figure 3a). Chronic CSE exposure did not change the percentage of ciliated cells until day 21 (Figure 2a upper panel and Figure 3a). In controls, after 28 days, approximately 50–55% of cells were ciliated. In contrast, chronic CSE exposure strikingly reduced ciliated cell numbers at this time point in a concentration-dependent manner (Figure 2a upper panel, Figure 2b left panel and Figure 3a).

Independent of CSE exposure, the fraction of CC10-positive Clara cells reached a peak of about 20% at day 14 (Figure 2a middle panel and Figure 3b). After that a gradual drop to about 10% at day 28 was observed under control culture conditions as well as when 2.5% CSE was included in the culture medium. In the presence of 5% CSE, Clara cell numbers did not decline between day 14 and day 28. Thus, Clara cell numbers were significantly greater in the presence of 5% CSE compared to control conditions at day 28 (Figure 2a middle panel, Figure 2b right panel and Figure 3b).

Goblet cells were quantified by positivity for MUC5AC (mucin 5AC). In control cells, goblet cells increased up to 21 days and declined afterwards (Figure 2a lower panel and Figure 3c). 5% CSE exposure significantly increased MUC5AC positive cells after 28 days (Figure 2a lower panel, Figure 2b right panel and Figure 3c). On average, 73.6% differentiated cells were quantified in control conditions at day 28, whereas this number dropped to 57.9% or 63.9% in 2.5% or 5% CSE-exposed cells, respectively. The percentages, however, need to be viewed with caution since double positive cells for MUC5AC and CC10 exist (Figure 2b)^24^. Overall, these data suggest that chronic CSE exposure specifically affects differentiation to ciliated cells between 21 and 28 days of differentiation at ALI, partly in favour of Clara and goblet cell fractions.

### CSE Alters pHBEC Differentiation in a Post-transcriptional Manner

**Figure 4:**
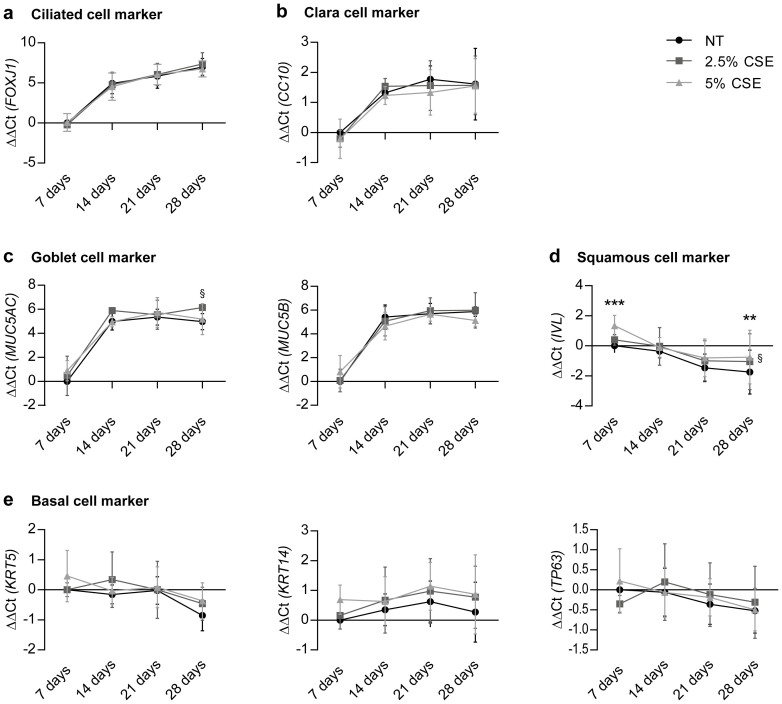
Chronic CSE exposure of pHBECs does not affect differentiation markers on mRNA level. qRT-PCR analysis of transcript levels of the ciliated cell marker *FOXJ1* (a), the Clara cell marker *CC10* (b), the goblet cell markers *MUC5AC* and *MUC5B* (c), squamous cell marker *IVL* (d) and the basal cell markers *KRT5*, *KRT14*, and *TP63* (e). pHBECs were differentiated up to 28 days in the absence or presence of CSE (2.5% or 5% CSE). Data are depicted as mean ± SD from 3 independent differentiations. Relative transcript abundance of a gene is expressed as ΔΔCt = [ΔCt(gene of interest, condition NT, day 7)] – [ΔCt(gene of interest, condition X, day X)] with ΔCt = Ct(gene of interest) – Ct (reference); increase = increase in gene expression. For 5% CSE vs. NT: **p < 0.01, ***p < 0.001. For 2.5% CSE vs. NT: ^§^p < 0.05 (two-way ANOVA).

**Figure 5:**
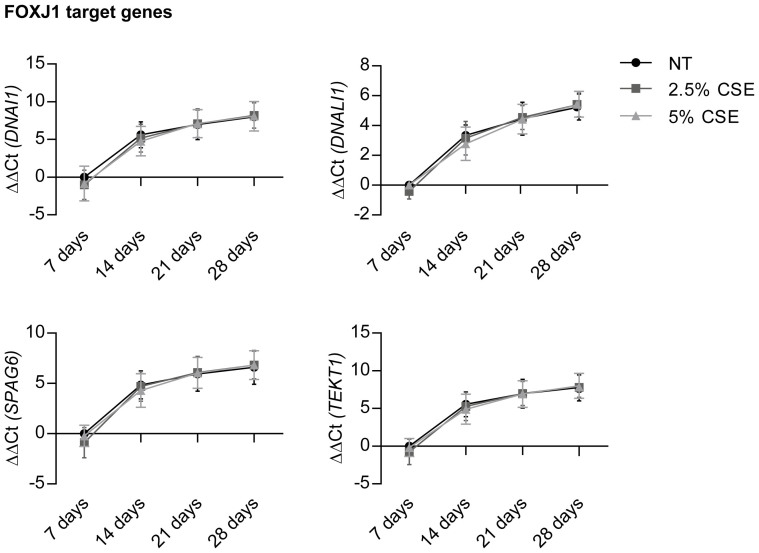
CSE does not alter transcript levels of FOXJ1 target genes. qRT-PCR analysis of transcript levels of the FOXJ1 target genes *DNAI1, DNALI1, SPAG6*, and *TEKT1*. pHBECs were differentiated up to 28 days in the absence or presence of CSE (2.5% or 5% CSE). Data are depicted as mean ± SD from 3 independent differentiations. Relative transcript abundance of a gene is expressed as ΔΔCt = [ΔCt(gene of interest, condition NT, day 7)] – [ΔCt(gene of interest, condition X, day X)] with ΔCt = Ct(gene of interest) – Ct (reference); increase = increase in gene expression.

**Figure 6:**
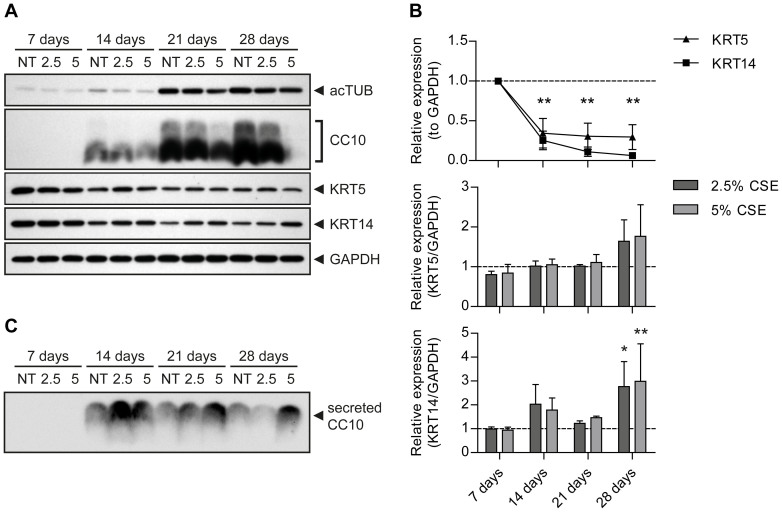
CSE alters protein expression of differentiation markers in pHBECs. (a) Western blot analysis of protein extracts from 7, 14, 21, or 28 days differentiated pHBECs either non-treated (NT) or treated for up to 28 days with CSE (2.5% or 5% CSE). Representative blots of acTUB (ciliated cell marker), CC10 (Clara cell marker), KRT5 and KRT14 (basal cell markers) and GAPDH as a loading control are shown. Protein samples were run on 8% and 15% gels under the same experimental conditions. Blots were cropped to improve clarity, full-length blots are presented in Supplementary Figure S2a. (b) Protein levels of KRT5 and KRT14 were quantified via densitometry analysis using Image Lab software. Data are depicted as mean ± SEM of four independent differentiations relative to GAPDH. Upper panel: In controls, KRT5 and KRT14 levels significantly decreased over time. **p < 0.01 vs. 7 days (one-way ANOVA). Middle and lower panel: KRT5 and KRT14 levels after CSE treatment at different time points. *p < 0.05, **p < 0.01 vs. NT at 28 days (two-way ANOVA). (c) Representative Western blot analysis for secreted CC10 in cell supernatants from 7, 14, 21, or 28 days differentiated pHBECs either non-treated (NT) or treated for up to 28 days with CSE (2.5% or 5% CSE) is shown. The blot was cropped to improve clarity, a full-length blot is presented in Supplementary Figure S2b.

To gain a better understanding of the mechanisms underlying the CSE-induced changes in pHBEC differentiation, we investigated the expression of different cell type specific markers on mRNA (Figure 4 and Figure 5) and protein (Figure 6) level. *FOXJ1* mRNA in control conditions increased greatly over 28 days (Figure 4a), thereby correlating well with the observed ciliated cell numbers (Figure 3a) and also with the detected protein levels of acetylated tubulin over time (Figure 6a and 6b). Interestingly, we did not find any alterations in *FOXJ1* mRNA expression, when cells were chronically exposed to CSE over 28 days (Figure 4a). In contrast, both concentrations of CSE decreased the levels of acetylated tubulin starting from day 14 after air-lift (Figure 6a and 6b), the time point when first motile cilia could be detected by immunofluorescence staining (Figure 2a upper panel). In order to evaluate whether FOXJ1 activity might be influenced by CSE, the expression of reported FOXJ1 target genes^27^ was measured (Figure 5). No significant alteration in *DNAI1*, *DNALI1*, *SPAG6*, and *TEKT1* transcripts compared to control conditions could be detected.

*CC10* mRNA increased up to day 21 after air-lift in control conditions, and decreased slightly afterwards (Figure 4b), correlating well with the quantified Clara cells numbers (Figure 3b). CSE stimulation had no influence on CC10 mRNA levels at either time point (Figure 4b). Of note, continuous CSE stimulation significantly decreased the amount of intracellular CC10 protein in a concentration-dependent manner starting from day 14 after air-lift (Figure 6a), while a concomitant increase of secreted CC10 could be detected in the cell supernatants of CSE-exposed cells (Figure 6c). Goblet cell numbers in control conditions (Figure 3c) were well mirrored by both *MUC5AC* and *MUC5B* mRNA (Figure 4c). In the presence of CSE, *MUC5AC* and *MUC5B* transcript levels were largely unchanged.

In order to evaluate the amount of basal cells present over time, expression of the transformation-related protein 63 (TP63), keratin 5 (KRT5), and keratin 14 (KRT14) was measured. In controls, *TP63, KRT5* and *KRT14* mRNA did not significantly change in the course of differentiation (Figure 4e) while protein levels for KRT5 and KRT14 significantly decreased over time (Figure 6a and 6b). Interestingly, continuous CSE stimulation was accompanied by a trend for increased KRT5 and KRT14 protein levels from day 21 after air-lift, a change which was significant for KRT14 at day 28 (Figure 6b). Finally, we also tested involucrin (*IVL*) expression over time (Figure 4d), a marker for squamous metaplasia e.g. in regions of basal cell hyperplasia. While *IVL* transcript levels decreased over time in control conditions, it was significantly elevated at day 7 and day 28 in the presence of CSE.

## Discussion

A functional airway epithelium is crucial for intact host defence against pathogens. In COPD, however, the epithelium is dramatically altered, which hampers pathogen clearance and triggers airway inflammation. Here we show that chronic exposure to CSE alters basal cell differentiation and function. We provide evidence that CSE specifically decreases ciliated cell numbers, while Clara and goblet cell numbers as well as basal cell marker expression were elevated in the presence of CSE.

Ciliated cells are the major cell type in the human trachea and bronchi, and normal coordinated ciliary beating is important for proper mucociliary clearance^28^. In the current study, we observed a striking difference of ciliated cell numbers in fully differentiated pHBECs after 28 days. While control cells reached about 53% ciliated cells, which is highly similar to the number of ciliated cells in the human tracheobronchial airway^1^, cells chronically exposed to CSE developed only about 30% ciliated cells. So far, areas of deciliation have only been reported for mice exposed to cigarette smoke^29^. Further, smoking has been associated with reduced cilia length^22^. In agreement with cilia shortening, we found a clear reduction in total acetylated tubulin upon CSE exposure at all time points, when motile cilia were present. Recent publications suggest cilia shortening to be autophagy-dependent^23^ or caused by CSE-dependent downregulation of structural cilia components and FOXJ^30^, a master regulator in ciliogenesis^31^. In contrast, we observed no changes in *FOXJ1* or FOXJ1 target gene transcription^27^ upon CSE exposure. This indicates that CSE influenced FOXJ1-independent processes crucial for ciliated cell fate, or affected ciliogenesis further downstream of FOXJ1.

It is evident that human basal cells possess stem cell capacity^8^ as they give rise to ciliated cells in ALI culture systems or sphere-forming assays. Apart from that, epithelial cell differentiation pathways from pluripotent basal cells to fully differentiated cells, partly via intermediate phenotypes, has so far only been studied in mouse models using lineage tracing^6^,^7^,^10^,^32^. In contrast, the nature of the human progenitor cells is largely unknown and, of note, major species differences exist in airway composition: Humans have a pseudostratified epithelium up to the terminal bronchioles with mainly ciliated, basal, and secretory cells (predominantly goblet cells). Only the trachea in mouse is pseudostratified with ciliated, basal, and secretory cells (mainly Clara cells, but only scarce goblet cells). The respiratory bronchioles in humans are lined by a columnar epithelium without basal cells, but consist of Clara and some ciliated cells. In mouse, however, the columnar epithelium starts already in the main bronchi with mainly ciliated and Clara cells and very few goblet cells but no basal cells. Mouse studies suggest the existence of stem cell populations in the intralobar bronchioles and the tracheobronchial region^32^. For instance, Clara cells have been shown to self-renew and to give rise to ciliated cells^32^. These Clara cells with stem cell-like characteristics are often referred to as variant Clara cell. Furthermore, basal cells in the murine trachea and main bronchi have been reported to differentiate in a Notch signalling-dependent manner where high Notch levels promote differentiation into secretory cells while low Notch levels possibly promote ciliated cell fate^33^. In addition, also so-called transit-amplifying cells (Cc10 negative) are thought to gradually replace Clara cells in the murine trachea and main bronchi^32^. Our data indicate that cigarette smoke hinders progenitor cells, including basal and possibly Clara cells, from entering the pathways of ciliated cell differentiation. It is likely that the resulting reduction in ciliated cell number along with a lower ciliary beat frequency contributes to reduced mucociliary clearance found in smokers and COPD patients^34^.

Clara cell numbers peaked at day 14 in all treatment conditions. While control cells gradually decreased to about 10% Clara cells until day 28, cultures exposed to 5% CSE exhibited about 17% Clara cells at this point, a difference that was statistically significant (p < 0.01). Clara cells may have progenitor cell potential as mentioned above, thus, this relatively small increase in Clara cells due to CSE may have far-reaching effects. This, however, remains to be elucidated. Interestingly, we found decreased levels of intracellular CC10 protein upon CSE treatment at different time points, but enhanced CC10 secretion. Since evidence from mouse studies suggests the existence of variant Clara cells mentioned above, which are reported to give rise to ciliated and goblet cells^32^, we hypothesize that trans-differentiation from Clara to ciliated cells might be impaired in the presence of CSE. As CC10 seems to fulfil an immunosuppressive function in the respiratory tract^11^,^12^, its enhanced secretion could reflect the increased need of anti-inflammatory mediators in the presence of smoke, i.e. to protect basal cell function. In contrast to our results, a reduction in Clara cell numbers has been found in smokers^35^ and smoke-exposed rats^36^, whereas no change in Clara cell numbers could be detected in smoke-exposed mice^37^. However, in agreement with our observations, enhanced CC10 serum levels correlate with the dysfunctional bronchial barrier in COPD patients and have therefore been suggested as a biomarker for disease progression^38^. The discrepancies between our study and others regarding Clara cell number changes following smoke exposure might reflect species-specific differences, limitations of our culture system such as basolateral exposure to CSE instead of cigarette smoke from the apical side, or the absence of a fully differentiated epithelium at the beginning of treatment.

Goblet cells are the major producers of mucus which captures inhaled particles and is cleared out by ciliary beating. In the presence of CSE, we observed a 7% increase in goblet cell numbers in fully differentiated pHBECs. This result fully agrees with the goblet cell hyperplasia found in (ex-) smokers with COPD^17^,^18^,^39^ or in smoke exposed rats^40^,^41^. Furthermore, CSE has also been demonstrated to induce mucus hypersecretion in various *in vitro* and *in vivo* models. From asthma research we know that Interleukin (IL)-13 plays a central role in the development of goblet cell hyperplasia^42^. Here we showed that smoke might also contribute to goblet cell hyperplasia found in COPD patients. Notably, the goblet cells could partly derive from FOXJ1-expressing progenitor cells, a trans-differentiation process recently reported in pHBEC^43^. Thus, the decreased ciliated cell number in spite of unchanged FOXJ1 expression found in our system could in part be explained by a smoke-dependent cell fate switch of FOXJ1-expressing cells towards the goblet cell phenotype.

We found an overall reduction in differentiated cells when pHBEC cultures were chronically exposed to CSE. This observation is reflected by an increase in basal cell specific protein expression (in particular KRT14) and an increase in squamous cells in our system, which suggests that basal cell differentiation is impaired by CSE. In agreement with our results, an expansion of TP63, KRT5, and KRT14 positive basal cells have been found in squamous metaplasia regions in airways of smokers with COPD^6^.

Taken together, our data suggest that the ALI model used here provides a suitable tool to study cellular airway epithelial changes found in COPD patients. We were able to recapitulate smoke-related changes, such as goblet and squamous metaplasia, and thus provide evidence that smoke directly affects basal cell differentiation. Moreover, we demonstrate that CSE reduces ciliated cell numbers in the bronchial epithelium via a FOXJ1-independent pathway or factors further downstream of FOXJ1. At the moment, studies on intermediate progenitor cells in the human epithelium are still in their infancy compared to mouse. Thus, in regard of the major species differences, our established human cell culture model is of high relevance and allows further mechanistic investigation of CSE-associated changes in the human bronchial epithelium.

## Methods

For a more detailed method section and standard methods, please refer to the online supplement.

### Cultivation, Differentiation and CSE Exposure of Primary Human Bronchial Epithelial Cells

Normal primary human bronchial epithelial cells (pHBECs) from two different donors (Lonza; Wokingham, UK) were routinely characterized for basal cell marker expression (Supplementary Figure S1) and cultured in BEGM medium (Lonza) as previously described^24^. pHBEC cultures were left to differentiate up to 28 days after air-lift. For CSE treatment during differentiation, cells were chronically exposed to 2.5 and 5% CSE between day 0 and day 28 of ALI culture from the basolateral side of the transwell (Figure 1a). CSE treatment was renewed every 2–3 days, i.e. each time the growth medium was changed. The apical surface was washed weekly with 0.5 ml pre-warmed HBSS to remove mucus. This solution (further referred to as “cell supernatant”), as well as the basal medium, was frozen at −80°C for analysis of secreted proteins.

### Preparation of CSE

100% CSE was generated as previously described^24^. Briefly, mainstream smoke of Research-grade cigarettes (3R4F) with filter (Kentucky Tobacco Research and Development Center at the University of Kentucky; Lexington, KY) was bubbled through 100 ml BEBM medium (Lonza) in a closed environment with a flow rate of 0.3 liter/minute. The burning time per cigarette was about 4 minutes. The obtained medium was considered as 100% CSE. To ensure standardization between experiments, CSE was sterile-filtered through a 0.2 μm filter (Minisart; Sartorius Stedim Biotech), aliquoted, and stored at −80°C. For usage, CSE was quickly thawed and diluted with BEGM medium to the indicated concentration.

### Transepithelial Electrical Resistance (TEER) Measurements

TEER development of differentiating pHBECs was monitored using a Millicell-ERS-2 (Millipore; Billerica, MA) volt-ohm-meter with a STX01 chopstick electrode (Millipore)^24^. For this, 500 μl pre-warmed HBSS was added to the apical compartment and left to equilibrate for 10 minutes in the incubator. Subsequently, a triplicate measurement was performed for every well. For different treatment conditions, 6 to 12 individual wells were analysed per experiment and time point. The values measured were corrected for blank values and area. For this, the average resistance of a blank well (without cells) was subtracted from the measured value of every well. The obtained value was multiplied by the effective membrane area in cm^2^ (1.12 cm^2^ for 12-well transwell inserts) to yield the final result in Ω x cm^2^. After TEER measurement, the apical solution was removed to restore ALI culture conditions.

### Cytotoxicity Assays

To assess cytotoxicity of CSE on cells, lactate dehydrogenase (LDH) levels were quantified in the cell supernatant and basolateral culture medium as described previously^24^. As a positive control for maximal LDH release, the supernatant of lysed cells was used.

### Immunofluorescence Analysis and Quantification

pHBECs were stained on the transwell membrane and the different cell fractions quantified as described previously^24^.

### Statistical Analysis

If not stated otherwise, data are depicted as mean ± SD and samples were harvested from three independently performed differentiations from two very different donors (donor #1: male, Hispanic, 61 years, used for 2 independent differentiations; donor #2: female, Asian, 62 years, used for 1 differentiation). For statistical analysis, repeated measures two-way ANOVA with Bonferroni post-tests versus the control condition (NT) was performed for most experiments. Protein levels of KRT5 and KRT14 over time in control conditions were analysed using one-way ANOVA with Bonferroni post-tests. Significance levels: *p < 0.05, **p < 0.01, ***p < 0.001.

## Author Contributions

O.E., N.M. and A.C.S. designed the study. A.C.S. acquired the data. A.C.S. and C.A.S. analysed the data. O.E., N.M., C.A.S. and A.C.S. discussed the results. A.C.S. drafted the manuscript. O.E., N.M. and C.A.S. critically revised the manuscript.

## Supplementary Material

Supplementary Information Online supplement
