# Supplementary material for: Cigarette smoke alters primary human bronchial epithelial cell differentiation at the air-liquid interface
Source: Sci Rep. 2015 Feb 2;5:8163. doi: 10.1038/srep08163 (PMC4313097; doi:10.1038/srep08163)

ONLINE DATA SUPPLEMENT

Cigarette smoke alters primary human bronchial epithelial cell differentiation at the air-liquid interface

Andrea C. Schamberger[1], Claudia A. Staab-Weijnitz[1], Nikica Mise-Racek[1], and Oliver Eickelberg[1,*]

[1]Comprehensive Pneumology Center, University Hospital, Ludwig-Maximilians-University and Helmholtz Zentrum München, Member of the German Center for Lung Research, Max-Lebsche-Platz 31, 81377 Munich, Germany

*To whom correspondence should be addressed: Oliver Eickelberg, Comprehensive Pneumology Center, University Hospital, Ludwig-Maximilians-University and Helmholtz Zentrum München, Member of the German Center for Lung Research, Max-Lebsche-Platz 31, 81377 Munich, Germany, Tel.: 0049(89)31874666; Fax: 0049(89)31874661; Email: oliver.eickelberg@helmholtz-muenchen.de

METHODS SECTION

Cultivation, differentiation and CSE exposure of primary human bronchial epithelial cells

Normal primary human bronchial epithelial cells in passage 1 (pHBECs) (Lonza; Wokingham, UK) were cultured in BEGM medium (Lonza) at 37°C in a humidified cell incubator with 95% air and 5% CO_2 [1]. When cell density reached 80%, cells were detached using the Clonetics™ ReagentPack™ (Lonza) for subculturing. For differentiation of HBECs, 80,000 - 90,000 cells at passage 2 were seeded onto pre-warmed human placental collagen type IV-coated (Sigma-Aldrich; St. Louis, MO) 12-well transwell inserts (transparent, 0.4 µm; Greiner; Solingen, Germany or clear, 0.4 µm; Corning; New York, NY) in pre-warmed BEGM medium. Cells were lifted to the air-liquid interface (ALI) when confluent, referred to as day 0 of ALI culture. For this, the apical medium was aspirated and the basolateral medium substituted with PneumaCult™-ALI medium (Stemcell Technologies; Köln, Germany) containing 1% penicillin/streptomycin. Medium was changed every 2 - 3 days. pHBEC cultures were left to differentiate up to 28 days after air-lift. For CSE treatment during differentiation, cells were chronically exposed to 2.5 and 5% CSE between day 0 and day 28 of ALI culture from the basolateral side of the transwell. CSE treatment was renewed every 2 - 3 days, i.e. each time the growth medium was changed. The apical surface was washed weekly with 0.5 ml pre-warmed HBSS to remove mucus. This solution (further referred to as "cell supernatant"), as well as the basal medium, was frozen at –80°C for analysis of secreted proteins.

Cytotoxicity Assays

To assess cytotoxicity of CSE on cells, lactate dehydrogenase (LDH) levels were quantified in the cell supernatant and basolateral culture medium using the Cytotoxicity Detection Kit (LDH) (Roche; Mannheim, Germany) according to the manufacturer's instructions. Briefly, supernatant and medium of untreated (= low control) and treated cells were collected. To control for maximal LDH release of the cells used (= high control), untreated cells were lysed with 2% Triton X-100/ media/0% FCS for 15 minutes at room temperature. Cell debris were pelleted with 250 x g for 10 minutes at 4°C and the cell-free liquids were stored at 4°C until analysis. Samples were mixed with equal amounts of substrate mixture and incubated for 30 minutes at room temperature in the dark. Absorbance (490 nm) was measured using the Sunrise multiplate reader, background values were subtracted, and cytotoxicity calculated using the formula:

$$Cytotoxicity\ (\%) = \frac{experimental\ value - low\ control}{high\ control - low\ control} \times 100.$$

RNA Isolation and Real-Time Quantitative Reverse-Transcriptase PCR (qRT-PCR) Analysis

For RNA extraction from pHBECs, the RNeasy Mini Plus Kit (Quiagen; Venlo, Holland) was used according to the manufacturer's instructions. RNA concentration was determined using the NanoDropTM 1000 spectrophotometer (NanoDrop Tech. Inc; Wilmington, DE) at 260 nm. For analysis of mRNA expression, total RNA was reverse transcribed using MuLV reverse transcriptase (Applied Biosystems; Carlsbad, CA) and random hexamer primers (Applied Biosystems). For this, 1 µg RNA was diluted up to 20 µl with DNase/RNase-free ddH_20. RNA secondary structures were denatured at 70°C for 10 minutes, before samples were incubated on ice for 5 minutes. 20 µl of cDNA synthesis master mix (5 mM $MgCl_2$, 1x PCR buffer II (10x), 1 mM dGTP, 1 mM dATP, 1 mM dTTP, 1 mM dCTP, 1 U/µl RNase inhibitor, and 2.5 U/µl MuLV reverse transcriptase) was added to each sample and cDNA synthesis was performed: 60 minutes at 37°C, followed by 10 minutes incubation at 75°C. cDNA was diluted up to 200 µl with DNase/RNase-free ddH_20 for usage in qRT-PCR analysis. qRT-PCR was performed in 96-well format using the Light Cycler LC480II instrument (Roche) and LightCycler® 480 DNA SYBR Green I Master (Roche). Relative transcript abundance of a gene is expressed as ΔΔCt = [ΔCt(gene of interest, condition NT, day 7)] – [ΔCt(gene of interest, condition X, day X)] with ΔCt = Ct(gene of interest) – Ct (reference). For specific gene amplification, primers listed in Table S1 were used. HPRT was used as a housekeeping gene for standardization of relative mRNA expression. All qRT-PCR reactions were performed in technical triplicates and non-template controls were used for quality controls.

Protein Isolation, SDS-Polyacrylamid Gel Electrophoresis (SDS-PAGE) and Immunoblotting

For whole cell lysates, cells were scratched into ice-cold RIPA lysis buffer (50 mM Tris•HCl, pH 7.6, 150 mM NaCl, 1% Nonidet P-40, 0.5% sodium deoxycholate, and 0.1% SDS) supplemented freshly with CompleteTM protease inhibitor cocktail (Roche) and PhosSTOP phosphatase inhibitor cocktail (Roche). For complete cell lysis, the collected cells were transferred to a microcentrifuge tube and incubated for 20 - 30 minutes on ice. Insoluble proteins were pelleted by centrifugation with 14,000 x *g* for 15 minutes at 4°C. The supernatant was transferred to a microcentrifuge tube and stored at -80°C until further analysis. Protein concentrations were determined using BCA™ Protein Assay Kit (Pierce Biotechnology Inc; Rockford, IL) according to the manufacturer's instructions.

Proteins were separated by SDS-PAGE according to Laemmli (1970) and transferred to polyvinylidene difluoride (PVDF) membrane according to Tobwin (1979). 8% and 15% SDS-gels were poured following the instructions in Current Protocols in Molecular Biology (Gallagher, 2001). Equal amounts of protein (15 - 30 µg) or equal volumes of cell supernatant were mixed with Laemmli loading dye and PBS for equal volumes and

denatured at 95°C for 5 minutes. Protein samples were separated by SDS-PAGE with constant 20 - 30 mA per gel in Laemmli running buffer, followed by wet protein transfer to methanol activated PVDF membrane with 330 mA for 60 minutes in transfer buffer.

5% (w/v) non-fat dry milk in TBS/0.2% (v/v) Tween-20 (TBST) was used as blocking agent for 1 hour at room temperature and TBST as washing agent. Detection of antigens was performed using the appropriate primary antibody in 5% (w/v) BSA in TBST, incubated overnight at 4°C while shaking. The membrane was washed in excess TBST at room temperature and subsequently incubated with the appropriate horseradish peroxidase (HRP)-conjugated secondary antibody in 5% (w/v) BSA in TBST for 1 hour at room temperature. After excess washing, antigens were visualized by enhanced chemiluminescence. Signals were documented with the ChemiDoc XRS$^+$ Imaging System (BioRad; Hercules, CA) or captured on X-ray film and developed in the AGFA Curix 60 processor (Siemens Healthcare; Erlangen, Germany). The following antibodies were used: mouse anti-acetylated tubulin (Sigma-Aldrich), mouse anti-CC10 (Santa Cruz; Santa Cruz, CA), rabbit anti-KRT5 and mouse anti-KRT14 (Abcam; Cambridge, MA), and HPR-conjugated anti-GAPDH (Cell Signaling; Danvers, MA).

Immunofluorescence Analysis and Quantification

pHBECs were stained directly on the transwell membrane, which was cut into quarters using a scalpel [1], or as cytospins Following the indicated cell treatment, cells were washed twice in DPBS and fixed in 3.7% (w/v) formaldehyde for 1 hour at room temperature. Cells were washed twice with DPBS and permeabilized with DPBS/0.1% Triton X-100 for 5 minutes at room temperature. 5% (w/v) BSA/0.2% (v/v) Tween-20 in DPBS served as blocking agent for 1 hour at room temperature. Detection of antigens was performed using the appropriate primary antibody in 5% (w/v) BSA/0.2% (v/v) Tween-20 in DPBS for 1 hour at room temperature. Subsequent to washing in excess DPBS, the cells were incubated with the appropriate secondary antibody conjugated with either Alexa Fluor 488 or Alexa Fluor 568 for 30 minutes at room temperature in the dark. The cellular DNA was stained with 0.5 µg/ml 4,6-diamidino-2-phenylindole (DAPI). Cells were washed in excess DPBS and mounted in fluorescent mounting medium (Dako; Hamburg, Germany) on microscopy slides using glass coverslips. The mounting medium was dried overnight at room temperature and fluorescent microscopy was performed using an upright microscope (Axiovert II; Carl Zeiss AG; Oberkochen, Germany) or confocal microscopy (LSM710 system; Carl Zeiss AG). Images were processed using ZEN 2010 software (Carl Zeiss AG) or Imaris 7.4.0 software (Bitplane; Zurich, Switzerland). Immunofluorescence quantification was performed using Imaris 7.4.0 software (Bitplane). For this, z-stack images of stained transwell membranes were obtained by

confocal microscopy and 1500 – 4500 cells per image were analyzed for positivity of specific markers in relation to the total number of cells assessed. Of note, also weakly expressing cells for specific markers were counted as positive signals. The following primary antibodies were used: mouse anti-acetylated tubulin (Sigma), rabbit anti-CC10 (Santa Cruz), and mouse anti-MUC5AC (Abcam).

Cytospin Analysis of pHBECs

In order to obtain cytospins from pHBECs, a single cell suspension of the cells was prepared using pre-warmed trypsin/EDTA from the apical side. Cells were pelleted at 250 x g for 5 minutes at room temperature and re-suspended in 1 ml HBSS. 30,000 to 100,000 cells in 200 µl HBSS were transferred to a glass slide using cytocentrifugation with 300 rpm for 10 min at room temperature. Cytospins were left to dry overnight at room temperature and processed immediately for immunofluorescent staining as described above.

TABLES

***Table S1.* Primers used for Real-Time Quantitative Reverse-Transcriptase Polymerase Chain Reaction**

Gene	***Forward primer (5'- 3')***	***Reverse primer (5'- 3')***
human *CC10*	TTCAGCGTGTCATCGAAACCC	ACAGTGAGCTTTGGGCTATTTTT
human *CYP1A1*	CTTGGACCTCTTTGGAGCTG	CGAAGGAAGAGTGTCGGAAG
human *DNAI1*	GAGTTGACCGATGCGGAGTT	TGTGCCTTCTTTGAAGCTGT
human *DNALI1*	CACCTCCAGGAGCAGTTAGAC	TGACCTCCCGGATCAACTCA
human *FOXJ1*	TCGTATGCCACGCTCATCTG	CTTGTAGATGGCCGACAGGG
human *HPRT*	AAGGACCCCACGAAGTGTTG	GGCTTTGTATTTTGCTTTTCCA
human *IVL*	GGAGGTCCCATCAAAGCAAGA	GCTCCTTCTGCTGTTGCTCA
human *KRT5*	GGAGTTGGACCAGTCAACATC	TGGAGTAGTAGCTTCCACTGC
human *KRT14*	TGGATCGCAGTCATCCAGAG	ATCGTGCACATCCATGACCT
human *MUC5AC*	AGCAGGGTCCTCATGAAGGTGGAT	AATGAGGACCCCAGACTGGCTGAA
human *MUC5B*	GCTGGAGCTGGATCCCAAAT	CTGGCGTTGTGGGCATAGA
human *SPAG6*	GCTGCCTTTGTGTTACGAGC	TCCAGTGCTCCACAATCGAC
human *TEKT1*	GAGAACGCCGTGAGGATTGA	AGCATCAGGGAGTTGTTCCG
human *TP63*	CCCGTTTCGTCAGAACACAC	CATAAGTCTCACGGCCCCTC

1 Schamberger, A. C. *et al.* Cigarette smoke-induced disruption of bronchial epithelial tight junctions is prevented by transforming growth factor-beta. *Am J Respir Cell Mol Biol* **50**, 1040-1052, doi:10.1165/rcmb.2013-0090OC (2014).

SUPPLEMENTARY FIGURE LEGEND

Supplementary Figure S1. **Marker characterization of basal cells used for differentiation.** (A) Representative indirect immunofluorescence analysis of cytospins of non-treated pHBECs at day 0. Cells are negative for differentiation markers, but positive for basal cell markers at day 0. (A) Representative staining for the basal cell markers p63 (mouse anti-p63, Santa Cruz), KRT5, and KRT14 is shown in green or red, as depicted, and DAPI staining in blue. Scale bar: 50 µm. (B) Representative staining for acTUB (ciliated cells), CC10 (Clara cells), and MUC5AC (goblet cells) is shown in green or red, as depicted, and DAPI staining in blue. Scale bar: 100 µm.

Supplementary Figure S2. **CSE alters protein expression of differentiation markers in pHBECs.** (A) Western blot analysis of protein extracts from 7, 14, 21, or 28 days differentiated pHBECs either non-treated (NT) or treated for up to 28 days with CSE (2.5% or 5% CSE). Full-length blots to Figure 6a of acTUB (ciliated cell marker), CC10 (Clara cell marker), KRT5 and KRT14 (basal cell markers) and GAPDH as a loading control are shown. Protein samples were run on 8 and 15% gels under the same experimental conditions. Signals were captured on X-ray film and developed in the AGFA Curix 60 processor. (B) Full-length blot to Figure 6b for secreted CC10 in cell supernatants from 7, 14, 21, or 28 days differentiated pHBECs either non-treated (NT) or treated for up to 28 days with CSE (2.5% or 5% CSE) is shown. Here, signals were documented with the ChemiDoc XRS^+ Imaging System and the merged image is displayed.

SUPPLEMENTARY FIGURES

Supplemental Figure S1

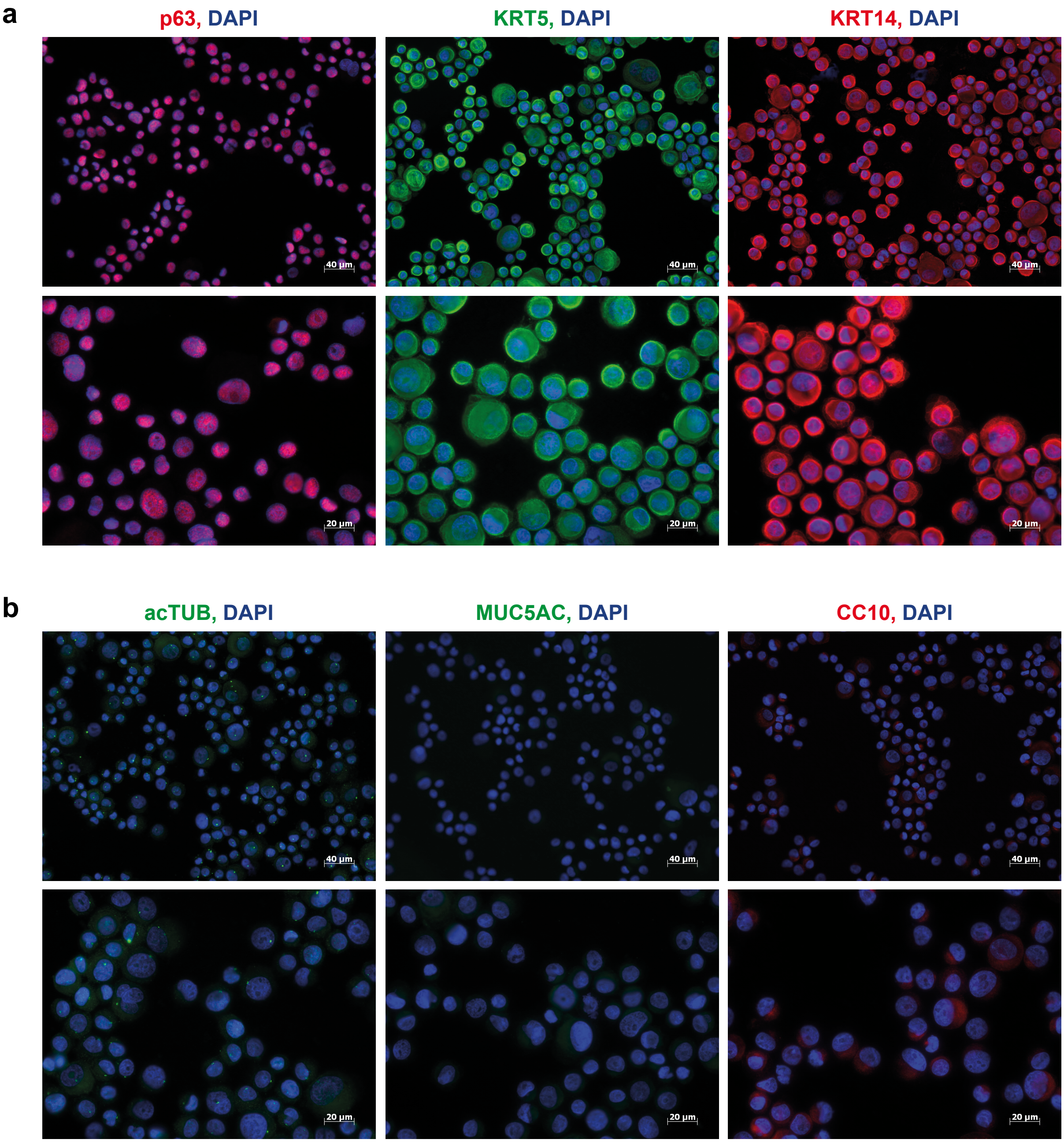

Supplemental Figure S2

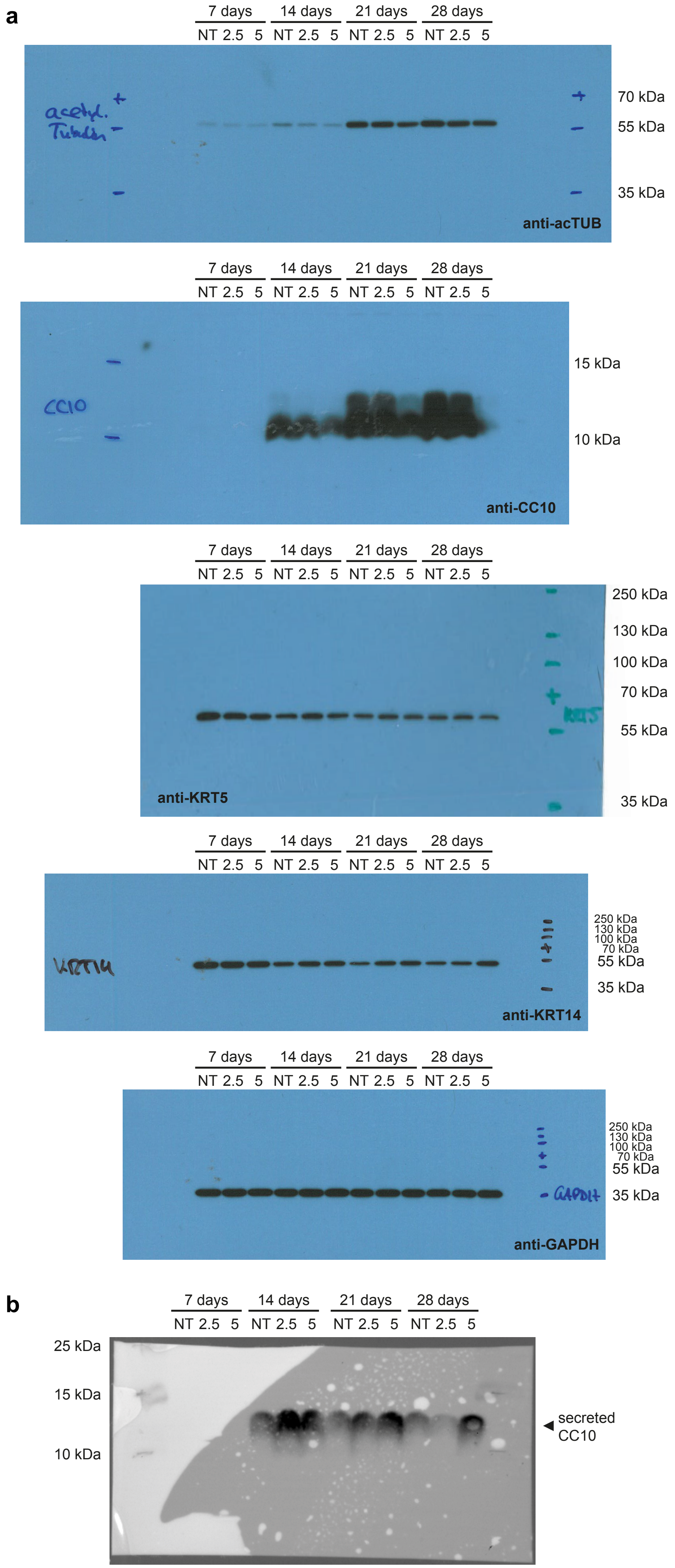

Supplement: Supplementary Information — Online supplement [file srep08163-s1.pdf]
